# Quantum memristors

**DOI:** 10.1038/srep29507

**Published:** 2016-07-06

**Authors:** P. Pfeiffer, I. L. Egusquiza, M. Di Ventra, M. Sanz, E. Solano

**Affiliations:** 1 Department of Physical Chemistry, University of the Basque Country UPV/EHU, Apartado 644, E-48080 Bilbao, Spain; 2 Department of Theoretical Physics and History of Science, University of the Basque Country UPV/EHU, Apartado 644, E-48080 Bilbao, Spain; 3 Department of Physics, University of California, San Diego, La Jolla, CA 92093, USA; 4 IKERBASQUE, Basque Foundation for Science, Maria Diaz de Haro 3, 48013 Bilbao, Spain

## Abstract

Technology based on memristors, resistors with memory whose resistance depends on the history of the crossing charges, has lately enhanced the classical paradigm of computation with neuromorphic architectures. However, in contrast to the known quantized models of passive circuit elements, such as inductors, capacitors or resistors, the design and realization of a quantum memristor is still missing. Here, we introduce the concept of a quantum memristor as a quantum dissipative device, whose decoherence mechanism is controlled by a continuous-measurement feedback scheme, which accounts for the memory. Indeed, we provide numerical simulations showing that memory effects actually persist in the quantum regime. Our quantization method, specifically designed for superconducting circuits, may be extended to other quantum platforms, allowing for memristor-type constructions in different quantum technologies. The proposed quantum memristor is then a building block for neuromorphic quantum computation and quantum simulations of non-Markovian systems.

Although they are often misused terms, the difference between information storage and memory is relevant. While the former refers to saving information in a physical device for a future use without changes, a physical system shows memory when its dynamics, usually named non-Markovian^1^,^2^, depend on the past states of the system. Recently, there is a growing interest in memristors, resistors with history-dependent resistance which provide memory effects in form of a resistive hysteresis^3^,^4^. In this sense, memristors, due to their memory capabilities^5^,^6^,^7^, offer novel applications in information processing architectures.

A classical memristor is a resistor whose resistance depends on the record of the electrical signals, namely voltage or charges, applied to it. The information about the electrical history is contained in the physical configuration of the memristor, summarized in its internal state variable *μ*, which enters the (voltage-controlled) memristor *I*-*V*-relationship via^8^,


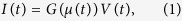



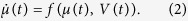


The state variable dynamics, encoded in the real-valued function *f*(*μ*(*t*), *V*(*t*)) and the state variable-dependent conductance function *G*(*μ*(*t*)) > 0, leads to a characteristic pinched hysteresis loop of a memristor under a periodic driving.

In superconducting circuits, electrical signals are quantized and can be used to implement quantum simulations^9^,^10^, perform quantum information tasks^11^, or quantum computing^12^,^13^. However, despite its prospects in classical information processing, memristors have not been considered so far in quantized circuits. The quantum regime of electrical signals is accessed by operating superconducting electric circuits at cryogenic temperatures. Their behavior is well-described by canonical quantization of Lagrange models that reproduce the classical circuit dynamics^14^. Furthermore, the effects of dissipative elements like conventional resistors are studied by coupling the circuit to environmental degrees of freedom represented by a transmission line^15^ or a bath of harmonic oscillators^16^. Yet, previous studies of memory elements in quantized circuits focussed only on non-dissipative components like memcapacitors and meminductors^17^,^18^,^19^. This is due to the fact that the history-dependent damping requires dissipative potentials, which cannot be cast in simple system-environment frames^20^.

In this Article, we propose a design of a quantum memristor (Fig. 1), closing the gap left by the classical memristor in the quantization toolbox for circuits^21^. Equation (2) can be understood as an information extraction process and, hence, its effect may be modeled on a circuit by continuous measurements. Furthermore, the state-dependent resistance in Eq. (1) is mimicked by a measurement-controlled coupling between the circuit and a bath of harmonic oscillators. Hence, our model for a quantum memristor constitutes a special case of quantum feedback control^22^, which is not restricted to superconducting circuits. As a paradigmatic example of a quantum memristor, we study a quantum LC circuit shunted by a memristor (see Fig. 2(a)), and address the compatibility between memory effects and quantum properties like coherent superpositions.

## Quantum memristor dynamics

We decompose the influence of a quantum memristor on the system into a Markovian tunable dissipative environment, a weak-measurement protocol, and a classical feedback controlling the coupling of the system to the dissipative environment. Then, the evolution of the circuit quantum state *ρ* includes a back action term due to the presence of the weak measurements. In addition, the dynamics contains a dissipative contribution, which depends on the state variable *μ*, and a Hamiltonian part, so that


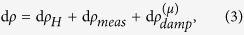


which essentially plays the role of Eq. (1). Correspondingly, if the interaction between the state variable and the circuit is cast in a measurement, the voltage records *M*_*V*_(*t*) govern the state variable dynamics,


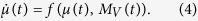


**Figure 2:**
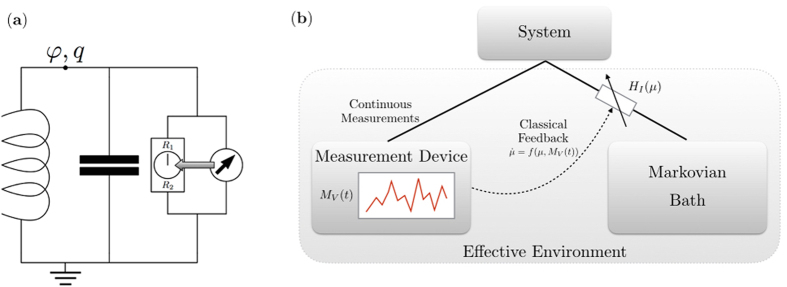
(**a**) Scheme of an LC circuit shunted by a memristor, which following our scheme, it is replaced by a tunable dissipative ohmic environment (depicted by the resistor with a knob to choose a resistance value between *R*_1_ and *R*_2_), a weak-measurement protocol (depicted by the voltmeter on the right), and a feedback tuning the coupling of the system to the dissipative environment depending on the measurement outcome (represented by the grey arrow). (**b**) Feedback model of a memristor and implementation in quantum dynamics via a feedback-controlled open quantum system.

Finally, the Hamiltonian part is determined by the circuit structure. In the following, we illustrate the method by shunting an LC circuit with a quantum memristor, as depicted in Fig. 2. Its description requires only one degree of freedom, the top node flux *φ*, and its conjugate momentum *q*^14^, which corresponds to the charge on the capacitor connected to the top node. Therefore, we have


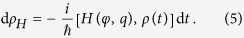


The measurement of the voltage applied to the quantum memristor implies a monitoring of the node charge *q*, since the voltage determines the charge on the capacitor with capacitance *C*, *q* = *CV*. Therefore, according to the theory of continuous measurements^22^,^23^, the state update and the measurement output have respectively the form


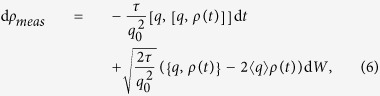



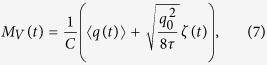


where {*A*, *B*} = *AB* + *BA* is the anticommutator and the mean value of an observable reads 〈*A*〉 = Tr(*ρ*A). The projection frequency *τ* is defined as the inverse of the measurement time needed to determine the mean charge up to an uncertainty *q*_0_, and depends on the measurement strength 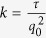. In the limit *τ* → ∞, we recover the usual projective measurement. On the other hand, in the limit *τ* → 0, the measurement apparatus is decoupled from the system, obtaining no information about it. Finally, the stochasticity of the measurement output enters via the white noise ζ(*t*), and the Wiener increment d*W* induces the corresponding probabilistic update of the quantum state.

A constant resistor with resistance *R* can be simulated by a bath of LC circuits with an ohmic spectral density^14^,


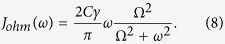


Here, the relaxation rate of the circuit is 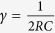 and Ω denotes a cut-off frequency. In the high temperature and high cut-off frequency limit, 
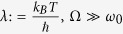
, with *ω*_0_ the typical circuit frequency, the dissipative contribution to the circuit dynamics can be cast in the Caldeira-Leggett (C-L) master equation^1^,^16^. In a sufficiently small time slice d*t*, in which *μ* remains approximately constant, a quantum memristor acts like a resistor with resistance *G*(*μ*)^−1^. Therefore, we adapt the C-L form by replacing the constant relaxation rate *γ* by a function 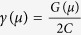,


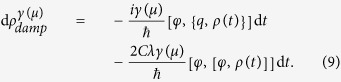


This phenomenological form could in principle be verified in a two-step procedure. Firstly, by capturing the full joint dynamics of the circuit and the bath when subjected to a feedback control of their interaction Hamiltonian. Secondly, by extracting the circuit dynamics after tracing out the bath degrees of freedom, as depicted in Fig. 2(b). However, the required feedback is non-Markovian, i.e. comprises voltage values from the past, and these systems are generally not analytically tractable^22^. Still, the C-L form represents a plausible choice, if the ordering *t*_*relax*_ ≪ *t*_*control*_ ≪ *t*_*exchange*_ ≪ d*t* holds for a time-coarse graining (See Supplementary material for a detailed argumentation).

Consider now an observer that has no access to the measurement output *M*_*V*_(*t*). From the observer’s point of view, the system evolves with density matrix 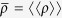, where 〈〈·〉〉 denotes the average over all realizations of the Wiener noise. We shall use the term *unconditioned state* to refer to 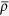. The evolution of the unconditioned state is determined by the ensemble average of Eq. (3). Clearly, we do not generally obtain a closed system for 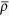 since 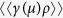, which appears in the ensemble average of the dissipative term, does not in principle factorize. Now, when it does factorize and 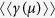 is constant, the evolution equation of 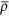 is of Lindblad form, describing a memoryless quantum Markov process. Thus, it provides us with a witness for non-Markovianity.

To sum up, the quantum-memristor dynamics given by increments of the quantum state in Eqs (5), (6) and (9), together with the state variable update in Eq. (4), evolves via two coupled, non-linear stochastic differential equations. Their form is designed to mimic the memory effect due to a memristor, by means of a damping rate which depends on partial information of the history of the quantum state. Unfortunately, this complex dynamics prevents an analytical approach, so we treat it numerically.

## Hysteresis in a quantum memristor

We present a numerical study of the dynamics of Gaussian states in an LC circuit shunted by a quantum memristor with linear state variable dynamics and the memristance of a Josephson junction^24^,^25^. For convenience, charge and flux are expressed in units of their vacuum fluctuations, 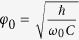 and 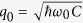, with *C* the capacitance. Furthermore, frequency is expressed in units of the circuit frequency 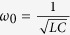, where *L* is the inductance of the coil. Hence, the Hamiltonian reads


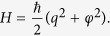


This quadratic Hamiltonian, as well as the damping and the measurement contribution to the dynamics, preserve the Gaussianity of an initial state. Therefore, it suffices to follow the evolution of the first and second moments of flux and charge (the charge and flux variances are defined as 
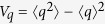
, 
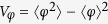
 and the covariance reads 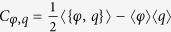). This, together with the damping function *γ*(*μ*) and the state variable dynamics, fully determine the dynamics of the conditioned state *ρ* (see ref. 23),


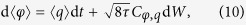



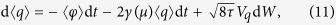



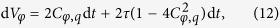



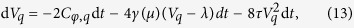



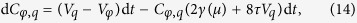



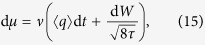



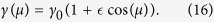


The frequency *ν* determines the rate of change of the state variable per unit of charge *q*_0_. If the unitless feedback parameter 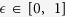 vanishes, the damping rate equals the constant *γ*_0_, and the system reduces to an LC circuit coupled to a constant resistor and a voltmeter. The specific form chosen for *γ*(*μ*) is inspired in Josephson junction physics (see ref. 25), but is only determined here for definiteness.

The set of Eqs (10)–(16) depends on the projection frequency *τ*, which is a free parameter, since it is not determined by Eqs (1)–(2), 2. Indeed, if we consider the classical limit corresponding to charging the capacitor for 〈*q*〉 → ∞, we recover Eqs (1)–(2), 2 for every positive *τ*. Therefore, there is an infinite family of quantum memristors producing the same classical memristive dynamics. However, in the low charging regime, the area of the hysteresis loop in the *I*-*V*-characteristic of the quantum memristor in the unconditioned evolution changes with *τ*. As the voltage is proportional to the charge on the capacitor and the conductance is proportional to the damping, to obtain the hysteresis loop means to plot 〈〈*V*〉〉 ∝ 〈〈*q*〉〉 vs. 〈〈*I*_*M*_〉〉 = 〈〈*γ*(*μ*)*q*〉〉. From the set of Eqs (10)–(16), two sources of diffusion of the state variable *μ* can be identified, namely, the noisy measurement output and the stochastic back-action on the first moments (terms in Equations (10)), and (15) proportional to the Wiener increment d*W*). These diffusive terms reduce the hysteresis area, since their physical origins, statistical averaging over multiple voltage histories and insufficient information extraction, respectively, counteract memory effects. Indeed, once the state variable is spread over a range ≥2*π*, the periodicity of the damping rate function leads to a stationary value of its ensemble average, 〈〈*γ*〉〉 = *γ*_0_, and the hysteresis loop collapses.

**Figure 3:**
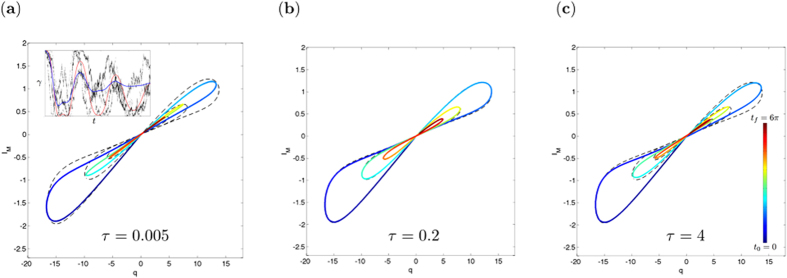
Hysteresis plots of the memristor for the unconditioned evolution with increasing projection frequencies *τ*. The comparison with the classical hysteresis curve (black, dashed) shows the collapse in the case of a very strong or very weak measurement. The inset shows the evolution of the damping rate and depict several of the underlying stochastic trajectories corresponding to one realisation of the conditioned dynamics. The parameters are 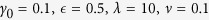, the initial conditions are 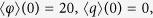

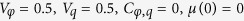
 and the average was obtained by generating 3000 trajectories of the stochastic dynamics via the Euler algorithm with d*t* = 10^−3^.

In Fig. 3, the successive collapse of the hysteresis for a strong and a weak measurement case is contrasted with the classical hysteresis, which almost coincides with the hysteresis for the optimal choose of *τ*, balancing information gain and measurement back-action (cf. Supplementary Material).

According to our model, the dynamics of the quantum LC circuit has acquired a non-Markovian character by coupling it to a quantum memristor. The question remains, whether characteristics of a genuine quantum system like coherent superpositions are affected by the memristive environment. The existence of non-linear terms in Eq. (10), which in principle destroy the coherence of the superpositions, makes non-trivial the answer to this question. In any case, one must understand this non-linear behavior as the *effective* action of the environment-measurement-feedback protocol, i. e. the quantum memristor, onto the system. This paves the way for employing these quantum memristors as a natural building block for simulating non-linear dynamics or designing non-linear dissipative gates. In particular, and in view of the success of the classical memristor proposal^5^,^6^, this opens the door to a possible development of neuromorphic architectures for quantum computing.

Similarly, one may wonder about the quantumness of the quantum memristor dynamics. Numerically, one can observe oscillations in the squeezing of the quantum state, so that an initial Gaussian state, whose variance is squeezed in momentum *V*_*q*_, periodically changes its squeezing to space, *V*_*φ*_, during the evolution. In other words, the system density matrix does not commute with itself for different times, which is an evidence of the quantumness of the dynamics^26^,^27^,^28^,^29^.

Even though the idea of engineering memristors in the quantum realm seems cumbersome, there are already proposals for employing the memristive component of the Josephson junctions in an asymmetric SQUID in superconducting qubits^24^. Unfortunately, the quantization of that proposed design is not complete, since it is described by a semiclassical model. More recently, a fully quantum realisation of a superconducting quantum memristor has been put forward^25^. This proposal exploits quasi-particle induced tunneling when supercurrents are cancelled in a Josephson junction, and the parameters explored there are achievable with current technology. This shows, at the very least, that a quantum memristor will soon be experimentally feasible.

## Conclusion

We have introduced quantum memristors and presented a protocol to construct properly the evolution equation for superconducting circuits coupled to quantum memristors. Our model is not restricted to electric circuits and could also be investigated in other quantum platforms like trapped ions or quantum photonics. Besides, we have constructively demonstrated the non-Markovian character of the quantum memristor dynamics, which allowed us to conjecture that the memory effects measured as the area of the hysteresis loop are maximized in the classical limit. Due to the impressive features shown by novel memristor-based computer architectures^5^,^6^,^30^, the quantum memristors proposed here may be considered as a building block for neuromorphic quantum computation and quantum simulation of non-Markovian systems.

## Additional Information

**How to cite this article**: Pfeiffer, P. *et al.* Quantum memristors. *Sci. Rep.* **6**, 29507; doi: 10.1038/srep29507 (2016).

## Supplementary Material

Supplementary Information

## Figures and Tables

**Figure 1:**
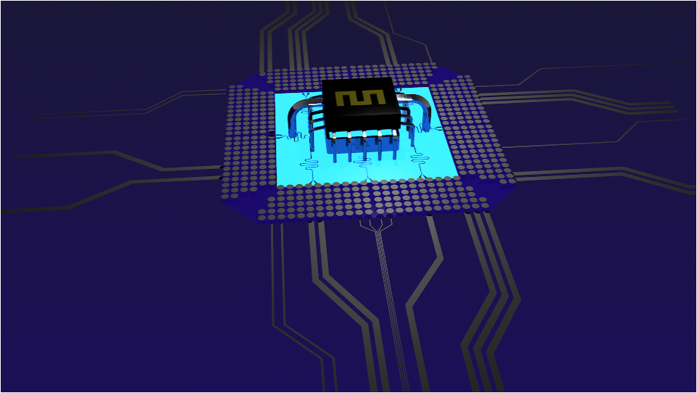
Artist view of a quantum memristor coupled to a superconducting circuit in which there is an information flow between the circuit and the memristive environment.

## References

[b1] Breuer H.-P. & Petruccione F. The Theory of Open Quantum Systems (Oxford University Press, 2007).

[b2] Gardiner C. Stochastic Methods: A Handbook for the Natural and Social Sciences (Springer: Berlin Heidelberg, , 2010).

[b3] Chua L. Memristor - The missing circuit element. IEEE Transactions on Circuit Theory 18, 507–519 (1971).

[b4] Strukov D. B., Snider G. S., Stewart D.R. & Williams R. S. The missing memristor found. Nature 453, 7191 (2008). 10.1038/nature06932 18451858

[b5] Traversa F. L. & di Ventra M. Universal memcomputing machines. IEEE Trans. Neur. Networks and Learn. Sys. 26, 2702 (2015). 10.1109/TNNLS.2015.2391182 25667360

[b6] Traversa F. L., Ramella C., Bonani F. & di Ventra M. Memcomputing NP-complete problems in polynomial time using polynomial resources and collective states. Sci. Adv 1, 5 (2015). 10.1126/sciadv.1500031 PMC4646770 26601208

[b7] Yang J. J., Strukov D. B. & Stewart D. R. Memristive devices for computing. Nature Nanotech. 8, 13–24 (2013). 10.1038/nnano.2012.240 23269430

[b8] Di Ventra M. & Pershin Y. V. On the physical properties of memristive, memcapacitive and meminductive systems. Nanotechnology 24, 255201 (2013). 23708238 10.1088/0957-4484/24/25/255201

[b9] Salathé Y. *et al.* Digital quantum simulation of spin models with circuit quantum electrodynamics. Phys. Rev. X 5, 021027 (2015).

[b10] Barends R. *et al.* Digital quantum simulation of fermionic models with a superconducting circuit. Nature Commun. 6, 7654 (2015). 26153660 10.1038/ncomms8654 PMC4510643

[b11] Felicetti S. *et al.* Dynamical Casimir effect entangles artificial atoms. Phys. Rev. Lett. 113, 093602 (2014). 25215982 10.1103/PhysRevLett.113.093602

[b12] Barends R. *et al.* Superconducting quantum circuits at the surface code threshold for fault tolerance. Nature 508, 500–503 (2015). 10.1038/nature13171 24759412

[b13] Ristè D. *et al.* Detecting bit-flip errors in a logical qubit using stabilizer measurements. Nature Commun. 6, 6983 (2015). 25923318 10.1038/ncomms7983 PMC4421804

[b14] Devoret M. H. In Quantum fluctuations, Lecture Notes of the 1995 Les Houches Summer School (eds Reynaud S., Giacobino E. & Zinn-Justin J. ), 351 (Elsevier, 1997).

[b15] Yurke B. & Denker J. S. Quantum network theory. Phys. Rev. A 29, 1419 (1984).

[b16] Caldeira A. O. & Leggett A. J. Quantum tunneling in a dissipative system. Annals of Physics 149, 374–456 (1983).

[b17] Di Ventra M., Pershin Y. V. & Chua L. O. Circuit elements with memory: Memristors, memcapacitors, and meminductors. Proceedings of the IEEE 97, 1717 (2009).

[b18] Di Ventra M. & Pershin Y. V. On the physical properties of memristive, memcapacitive and meminductive systems. Nanotechnology 24, 255201 (2013). 23708238 10.1088/0957-4484/24/25/255201

[b19] Shevchenko S. N., Pershin Y. V. & Nori F. Qubit-based memcapacitors and meminductors. ArXiv:1602.07230 (2016).

[b20] Cohen G. Z., Pershin Y. V. & di Ventra M. Lagrange formalism of memory circuit elements: Classical and quantum formulations. Phys. Rev. B 85, 165428 (2012).

[b21] Pfeiffer P. In Master’s Thesis: *Quantum memristors*, (Ludwig Maximilian University Munich, 2015).

[b22] Wiseman H. M. & Milburn G. J. Quantum measurement and control (Cambridge University Press, 2010).

[b23] Jacobs K. & Steck D. A. A straightforward introduction to continuous quantum measurement. Contemporary Physics 47, 279 (2006).

[b24] Peotta S. & di Ventra M. Superconducting memristors. Phys. Rev. Applied 2, 034011 (2014).

[b25] Salmilehto J., Deppe F., di Ventra M., Sanz M. & Solano E. Quantum memristors with superconducting circuits. ArXiv: 1603.04487 (2016). 10.1038/srep42044 PMC5307327 28195193

[b26] Sanz M., Pérez-García D., Wolf M. M. & Cirac J. I. A quantum version of Wielandt’s inequality. IEEE Trans. Inf. Theory 56, 4668 (2010).

[b27] Iyengar P., Chandan G. N. & Srikanth R. Quantifying quantumness via commutators: an application to quantum walk. ArXiv:1312.1329 (2013).

[b28] Ferro L. *et al.* Measuring quantumness: from theory to observability in interferometric setups. ArXiv:1501.03099 (2015).

[b29] Jing J., Wu L.-A. & del Campo A. Fundamental Speed Limits to the Generation of Quantumness. ArXiv:1510.01106 (2015). 10.1038/srep38149 PMC5128863 27901118

[b30] Simonite T. *IBM making plans to commercialize its brain-inspired chip*, MIT Technology Review (October 2015). Available at: http://www.technologyreview.com/news/542366/ibm-making-plans-to-commercialize-its-brain-inspired-chip/ (Retrieved: 6th November 2105).

