# Supplementary material for: Quantum memristors
Source: Sci Rep. 2016 Jul 6;6:29507. doi: 10.1038/srep29507 (PMC4933948; doi:10.1038/srep29507)
Supplement: Supplementary Information [file srep29507-s1.pdf]

SUPPLEMENTAL MATERIAL
"Quantum Memristors"

P. Pfeiffer,[1] I. L. Egusquiza,[2] M. Di Ventra,[3] M. Sanz,[1] and E. Solano[1,4]

[1]*Department of Physical Chemistry, University of the Basque Country UPV/EHU, Apartado 644, E-48080 Bilbao, Spain*
[2]*Department of Theoretical Physics and History of Science,*
University of the Basque Country UPV/EHU, Apartado 644, E-48080 Bilbao, Spain
[3]*Department of Physics, University of California, San Diego, La Jolla, CA 92093, USA*
[4]*IKERBASQUE, Basque Foundation for Science, Maria Diaz de Haro 3, 48013 Bilbao, Spain*

In this Supplemental Material, we discuss details useful for the understanding of the main results of the paper.

I. TIME SCALES OF THE QUANTUM MEMRISTOR DYNAMICS

The Caldeira-Leggett (C-L) model describes the dynamics of open quantum systems weakly coupled to a bath of harmonic oscillators at high temperature. Hence, it assumes, first, that the collection of harmonic oscillators is in a thermal state, and, second, that the interaction with the open system is governed by a fixed, weakly interacting Hamiltonian H_I [1].

In the model of the quantum memristor, the interaction Hamiltonian is controlled by measurement results. The output of measurements is included in Eq. (5) of the manuscript:

$$\begin{aligned} \mathrm{d}\rho_{meas} &= -\frac{\tau}{q_0^2}\left[q,\left[q,\rho(t)\right]\right]\mathrm{d}t \\ &\quad+\sqrt{\frac{2\tau}{q_0^2}}\left(\{q,\rho(t)\}-2\left\langle q\right\rangle\rho(t)\right)\mathrm{d}W, \qquad (1a)\end{aligned}$$

$$M_V(t) = \frac{1}{C}\left(\langle q(t)\rangle+\sqrt{\frac{q_0^2}{8\tau}}\zeta(t)\right). \qquad (1b)$$

There is a measurement result for every interval $\mathrm{d}t$. We assume systematically that the control time $t_{control}$ that is required at each step to fully achieve the necessary tuning of the interaction Hamiltonian is small when compared to the interval $\mathrm{d}t$. Schematically, the interaction Hamiltonian does not change during intervals of length $\mathrm{d}t$ and does change during intervals of width $t_{control}$, as illustrated in Fig. 1.

We require that during most of the time interval of length $\mathrm{d}t$ the bath, and the system interaction with it, be properly described by the C-L model. As a first consequence, we need the thermal structure of the harmonic oscillator bath to be guaranteed. Therefore, the bath relaxation time scale must be shorter than the control time,

$$t_{relax} \ll t_{control}.$$

Secondly, the interaction between the system and the bath has to be determined by the values of the interaction Hamiltonian in the plateaus, and should not depend on the form of the control step. Specifically, the time during which there is exchange of excitations between open system and bath needs to be large compared to the control time,

$$t_{control} \ll t_{exchange}.$$

In this case, no excitation is exchanged during the tuning of the interaction Hamiltonian.

In summary, our working assumption is the time scale ordering shown in Fig. 1,

$$t_{relax} \ll t_{control} \ll t_{exchange} \ll \mathrm{d}t.$$

II. CLASSICAL HYSTERESIS AND OPTIMAL PROJECTION FREQUENCY

Classical hysteresis

The classical hysteresis curve is obtained by treating the LC circuit coupled to the quantum memristor (see Fig. 2 of the manuscript) classically. The second Kirchhoff's law requires the currents from the three branches, I_L (inductor), I_C (capacitor) and I_M (quantum memristor) to sum to zero, $I_L + I_C + I_M = 0$, or equivalently,

$$\frac{\Phi}{L} + \dot{Q} + G(\mu)\frac{Q}{C} \;=\; 0. \tag{2}$$

Here, the inductor flux is Φ, the charge on the capacitor is denoted by Q, and the memconductance G is a function of the state variable μ. Furthermore, the voltages across the inductor and the capacitor are the same,

$$\dot{\Phi} = \frac{Q}{C}. \tag{3}$$

Finally, the second quantum memristor equation (see Eq. (1b) of the manuscript) determines the evolution of the state variable μ, which in our case is a linear function of the applied voltage

$$\dot{\mu} = \nu\frac{V}{V_0} = \nu\frac{Q}{Q_0}. \tag{4}$$

Here, the role of the memory frequency ν becomes clear. It determines the rate of change of the quantum memristor state variable per unit charge Q_0 on the capacitor. Clearly, it depends on the choice of this unit charge and we chose the charge fluctuation $(\times\sqrt{2})\ q_0 = \sqrt{\hbar\omega_0 C}$ of the LC-circuit in the ground state.

In the dimensionless notation used in the main body of the paper, equations (2) , (3) and (4) read

$$\begin{aligned}
\frac{\mathrm{d}}{\mathrm{dt}}\,q(t) &= -\varphi(t) - 2\gamma(\mu(t))q(t),\\
\frac{\mathrm{d}}{\mathrm{dt}}\,\varphi(t) &= q(t),\\
\frac{\mathrm{d}}{\mathrm{dt}}\,\mu(t) &= \nu q(t).
\end{aligned}$$

On solving this set of equations, the resulting V-I_M curve shows hysteresis in the voltage-current relation of the memristor for a classical circuit. The black dotted hysteresis curves in the q-$\gamma(\mu)q$ plot in Fig. 3 of the manuscript correspond to such solutions, with initial conditions given by the initial expectation values of the corresponding quantum operators $q(0) = \langle q\rangle(0)$ and $\varphi(0) = \langle\varphi\rangle(0)$, and the same initial state variable value $\mu(0) = \mu_0$.

Optimal projection frequency

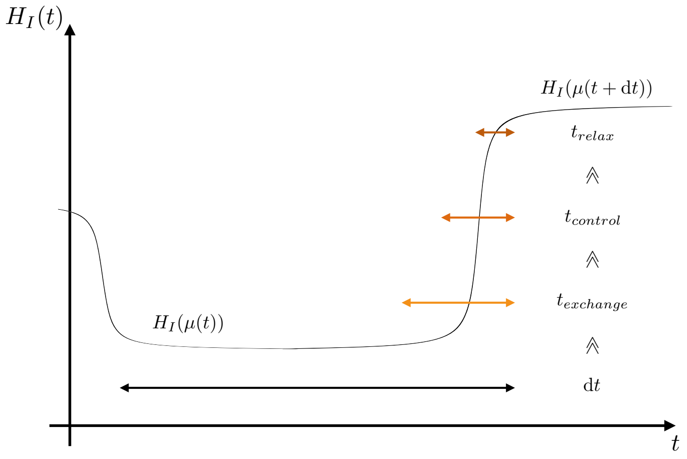

FIG. 1: Schematic tuning of the interaction Hamiltonian between bath and system. The time ordering of bath relaxation, control time and excitation exchange duration ensures the validity of the Caldeira-Leggett model in every interval $\mathrm{d}t$ of the coarse graining of time.

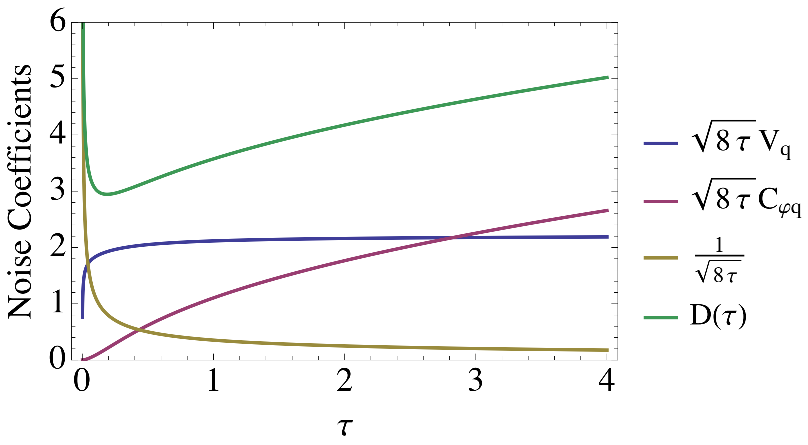

FIG. 2: Dependence of all diffusive terms in first moments and state variable on the projection frequency τ. The low noise regime suggested by this image is determined by $\tau \approx 0.2$, for the values of damping rate $\gamma = 0.1$ and thermal frequency $\lambda = 10$.

In our model τ, which is the projection frequency of the measurement, is a free parameter. It measures the rate in which information is extracted by measurements in the circuit. In a physical implementation of the feedback scheme in the quantum memristor model, it would be tuned by controlling the coupling between the open system and the measurement device. In the classical limit, which corresponds to a large charge in the capacitor, the effective classical memristor equations are independent of τ.

As discussed in the main text, the projection frequency determines the information gain about the circuit variables as well as their perturbation due to the measurement. Both uncertainty in the measurement, on the one hand, and backaction, on the other, lead to diffusion of the state variable. Since the projection frequency impacts on both, an optimal τ would correspond to a measuremen, which extracts sufficient information, while inducing only small fluctuations. In other words, the optimal τ minimises the state variable variance growth.

To determine an approximation of the optimal τ we analyse the sum of the noise terms in Eqs. (8) in the article,

$$D(\tau) = \sqrt{8\tau} V_q + \sqrt{8\tau} |C_{\varphi,q}| + \frac{1}{\sqrt{8\tau}}. \tag{5}$$

The charge variance V_q and the covariance $C_{\varphi,q}$ are a function of time, but they quickly reach quasi stationary values. There are small variations due to the changes in the damping rate γ. If we fix the damping rate at its mean value γ_0 (see Eq. (8g)), the stationary values of Eq. (8d) and (8e) are

$$C^{st}_{\varphi,q} = -\frac{1}{8\tau}\left(\sqrt{1+(4\tau)^2} - 1\right), \tag{6}$$

$$V^{st}_q(\tau) = \frac{\sqrt{\gamma_0^2 + 4\tau(2\gamma_0\lambda - C_{\varphi,q})} - \gamma}{4\tau}. \tag{7}$$

Inserting these values in Eqs. (8) for the parameters used in our simulations, the minimisation of the noise sum yields $\tau_{opt} \approx 0.2$ (see Fig. 2).

III. QUANTUM HYSTERESIS IS NOT PINCHED AT 0

In the unconditioned evolution of the quantum memristor, the $\langle\langle q \rangle\rangle$ vs. $\langle\langle \gamma(\mu) q \rangle\rangle$ curve is not necessarily pinched at the origin. In fact, numerically evaluating the deviation from factorisation $\delta_q = \langle\langle \gamma(\mu) q \rangle\rangle - \langle\langle \gamma(\mu) \rangle\rangle \langle\langle q \rangle\rangle$, provided small ($\leq 0.1$), but non-zero values for the zero crossings of $\langle\langle q \rangle\rangle$. The resulting hysteresis observed in the unconditioned evolution is depicted in Fig. 3. Due to the correlations between damping rate and charge on the capacitor, this corresponds to non-zero (quantum average) current for vanishing (quantum average) voltage. The passivity of the device is still guaranteed, as the expectation value of the emitted power fulfils $\langle\langle I_M V \rangle\rangle \propto \langle\langle \gamma(\mu) q^2 \rangle\rangle \geq 0$.

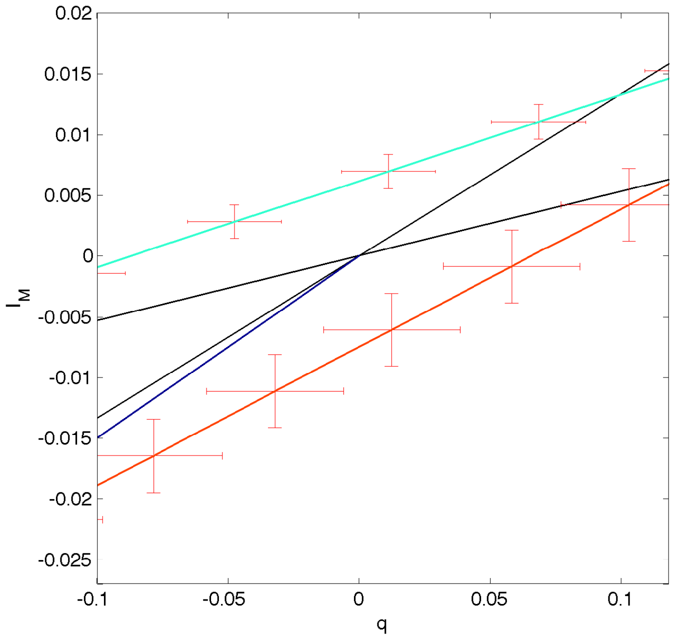

FIG. 3: Blow-up of the region around the origin of Fig. 3a of the article revealing non pinching at the origin.This is due to the deviation $\delta_q(t)$ from factorization of $\langle\langle\gamma(\mu)q\rangle\rangle$. Only one and a half oscillations are shown, for clarity. The black line corresponds to the classical I-V curve. The red bars denote the ensemble estimation error, not including numerical errors due to the Euler Algorithm.

IV. NOISY SIGNALS AND HYSTERESIS

Both noisy voltage signals and fluctuations of the state variable have been studied for classical memristors [2, 3]. However, for a classical voltage signal, these two noise sources are independent and can be simultaneously suppressed, whereas in the quantum case of the measurement back-action ties them together. The parameter that controls the noise amplitude is the measurement strength k, or more precisely, the projection frequency $\tau = q_0^2 k$, as defined in the main text. For a strong measurement, (large $\tau \gg 1/\omega_0$), noise in the state variable is suppressed, but the back-action increases the fluctuations in the first moments of flux and charge, thus leading to diffusion of the state variable. On the contrary, for a weak measurement, $\tau \ll 1/\omega_0$, first moments evolve in an almost unperturbed manner, but the loss of information in the voltage measurement produces a highly fluctuating state variable update. Finally, the accumulated noise has to be compared to the typical charge on the capacitor, q_t, which represents the signal to be recorded, and the threshold 2π, from which on hysteresis collapses. As a Wiener noise contribution of the form $D\mathrm{d}W$ induces a linear growth in time of the variance proportional to Dt^2, the condition for the existence of hysteresis up to a time t_C reads (in adimensionalized terms)

$$8\tau V_q t_C, 8\tau C_{\varphi,q} t_C, \frac{t_C}{8\tau} \ll \min(q_t^2, 4\pi^2). \tag{8}$$

Like all open quantum systems, a quantum memristor is subject to decoherence, a process which has been extensively studied in the context of the quantum-to-classical transition [4]. However, a memory-specific feature of the memristive environment consists in the continuous monitoring of the system with a fine-tuned measurement strength. It is known that continuous measurements are able to provide well-localised trajectories in phase space, a characteristic of classical systems [5]. In a linear system, such as the LC circuit, the measurement strength has to fulfil two conditions in order for localisation of trajectories to take place, namely, sufficient suppression of the variances and a negligible measurement back action in comparison to the system dynamics. The amplitude of the system dynamics is given by the typical action of the system s (in units of $\hbar$) and constraints the projection frequency by [6, Eq. (6)],

$$\frac{2}{s} \ll \tau \ll 4s. \tag{9}$$

The typical action can be estimated by $s = \frac{\langle E\rangle}{\hbar\omega_0} \approx q_t^2$ (with charge unit q_0) and therefore the conditions in Eq. (8) for a memory effect coincide with the requirement for a well-localised trajectory. Loosely speaking, a localised phase space trajectory keeps the state variable localised as well, and thus allows for memory effects.

In order to connect the requirement for a memory effect up to time t_C in Eq. (8) and the condition for a well-localised phase space trajectory in Eq. (9), we first concentrate on fluctuations of first moments. The noise in the first moments induced by the measurement becomes important for large measurement strengths, say $\tau > 1/\omega_0$. In this regime, and using the adimensional setting of the main text, the noise is dominated by the term $\sqrt{8\tau}C_{\varphi,q}$ with $C_{\varphi,q} \approx \frac{1}{2}$ and hence the first two inequalities in Eq. (8) require

$$\frac{8\tau t_C}{2} \ll q_t^2.$$

This corresponds to the upper bound for the projection frequency in Eq. (9) growing linearly with the typical action s in units of the planck constant, because $s = \frac{\langle E\rangle}{\hbar\omega} = \frac{q_0^2}{\hbar\omega C}q^2 = q^2$. Correspondingly, the last inequality in Eq. (8)

$$\frac{t_C}{8\tau} \ll q_t^2 \Rightarrow \frac{t_C}{8q_t^2} \ll \tau \tag{10}$$

provides the inverse scaling with the typical action s of the lower bound on τ in Eq. (9).

V. NUMERIC IMPLEMENTATION

We study Gaussian state dynamics in an LC circuit coupled to a quantum memristor with numerical simulation of the set of Eqs. (8) in the manuscript. These are stochastic differential equations with Gaussian noise and are cast in Itô form. We use the explicit Euler algorithm [7]. It is based on the discretisation of the equations according to

$$\begin{aligned} \mathrm{d}t &\rightarrow \Delta t, \\ \mathrm{d}W &\rightarrow G\sqrt{\Delta t}, \end{aligned}$$

where Δt has to be chosen sufficiently small and the G are independently, identically, distributed values drawn from a Gaussian distribution with mean 0 and variance 1.

Concretely, the simulation is implemented in Matlab (code available upon request). All the above results are obtained with a time increment $\Delta t = 10^{-3}$, which in the chosen units is much smaller than the period of one oscillation in the LC circuit, $T = 2\pi$. In order to obtain the hysteresis curves of the ensemble we average over 3000 trajectories.

The stability of our implementation with respect to the choice of the time increment and the sufficient ensemble size is demonstrated by a comparison of the evolution of the damping rate $\gamma(\mu(t))$ for different time increments in Fig. 4. Furthermore, we have also carried out a simulation using the inbuilt version of the Euler algorithm in Mathematica, and, qualitatively, the behaviour is the same. In fact our implementation is more stable for longer times, because of the poor handling of large ensemble sizes in the version of Mathematica available to us.

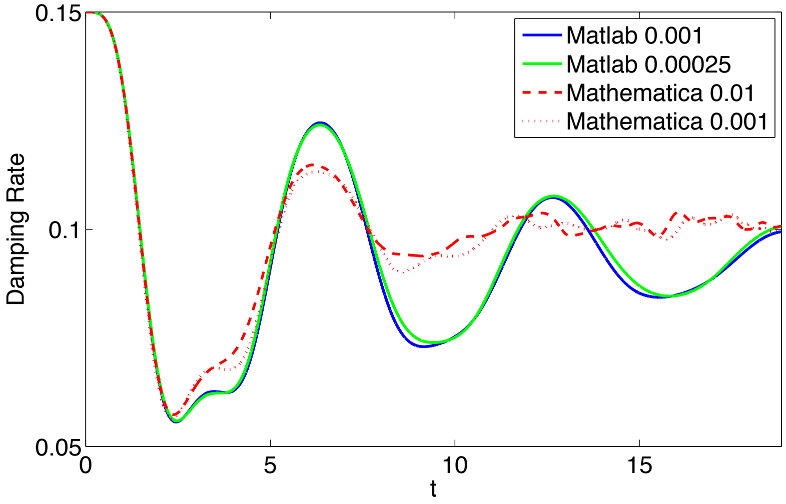

FIG. 4: Test of the Matlab code via a comparison of the evolution of the mean value damping rate with a solution obtained in Mathematica. The different lines depict different time increments $\mathrm{d}t$. The ensemble sizes are: 3000 (Matlab), 300 (Mathematica).

[1] Breuer, H.-P. & Petruccione, F. The Theory of Open Quantum Systems (Oxford University Press, 2007).

[2] Di Ventra, M. & Pershin, Y. V. On the physical properties of memristive, memcapacitive and meminductive systems. *Nanotechnology* **24**, 255201 (2013).

[3] Stotland, A. & di Ventra, M. Stochastic memory: Memory enhancement due to noise. *Phys. Rev. E* **85**, 011116 (2012).

[4] Schlosshauer, M. The quantum-to-classical transition and decoherence. arXiv:1404.2635 (2014).

[5] Jacobs, K. & Steck, D. A. A Straightforward introduction to continuous quantum measurement. *Contemporary Physics* **47**, 279 (2006).

[6] Bhattacharya, T., Habib, S. & Jacobs, K. Continuous quantum measurement and the emergence of classical chaos. *Phys. Rev. Lett.*, **85** 4852–4855 (2000).

[7] Gardiner, C. Stochastic Methods: A Handbook for the Natural and Social Sciences (Springer Berlin Heidelberg, 2010).
